# A pilot study of camrelizumab with docetaxel and cisplatin for the first line treatment in recurrent/metastatic oral squamous cell carcinoma

**DOI:** 10.1002/mco2.312

**Published:** 2023-07-22

**Authors:** Houyu Ju, Dongliang Wei, Yunteng Wu, Yang Liu, Qi Ding, Mengyu Rui, Zongyu Fan, Yanli Yao, Jingzhou Hu, Guoxin Ren

**Affiliations:** ^1^ Department of Oral Maxillofacial‐Head and Neck Oncology Shanghai Ninth People's Hospital Shanghai Jiao Tong University School of Medicine Shanghai China; ^2^ Shanghai Key Laboratory of Stomatology & Shanghai Research Institute of Stomatology National Clinical Research Center of Stomatology Shanghai China; ^3^ National Clinical Research Center of Stomatology Shanghai China; ^4^ School of Stomatology Weifang Medical University Weifang China

**Keywords:** camrelizumab, first‐line treatment, immunotherapy, oral cancer

## Abstract

Pembrolizumab with cisplatin and 5‐fluorouracil showed survival benefit but relatively high occurrence of treatment‐related adverse events (TRAEs) for recurrent/metastatic oral squamous cell carcinoma (R/M OSCC). A more tolerable regime is needed. This trial enrolled 20 R/M OSCC patients with previously untreated and PD‐L1 positive. Patients were administered camrelizumab with docetaxel and cisplatin every 3 weeks for six cycles, followed by camrelizumab monotherapy every 3 weeks until disease progression or intolerable toxicity. The primary endpoint was occurrence of grade ≥ 3 TRAEs, secondary endpoints included overall survival (OS), progression‐free survival (PFS), and overall response rate (ORR). 45% patients experienced grade ≥ 3 TRAEs, which the most common were anemia (15%), stomatitis (15%), and neutropenia (10%). The most common potential immune‐related adverse events were reactive cutaneous capillary endothelial proliferation (RCCEP; 60%), hypothyroidism (35%), and pneumonitis (15%). No treatment‐related deaths occurred. The median OS, PFS, and ORR was 14.4 months, 5.35 months, and 40.0% respectively. The study also found RCCEP occurrence, lower FOXP3^+^ cells, and higher density of intratumor tertiary lymphoid structure were associated with improved efficacy. Our data suggest that camrelizumab with docetaxel/cisplatin as first‐line therapy was well tolerable and had potentially favorite efficacy in PD‐L1‐positive patients with R/M OSCC.

## Graphical abstract

This phase Ib trial enrolled patients diagnosed recurrent/metastatic oral squamous cell carcinoma (R/M OSCC) with previously untreated and PD‐L1 positive. Our data suggest that camrelizumab with docetaxel/cisplatin was well tolerable and had favorite efficacy in PD‐L1‐positive patients with R/M OSCC. The study also found the occurrence of reactive cutaneous capillary endothelial proliferation, lower expression of FOXP3, and density of intratumor tertiary lymphoid structure were associated with better efficacy.

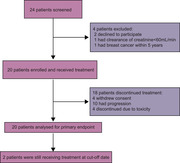

## 1 INTRODUCTION

Oral squamous cell carcinoma (OSCC) is frequent subtype malignancies in the head and neck region.^1^ Despite undergoing curative surgery, radiotherapy, and/or chemotherapy, patients with OSCC have a relatively high incidence of recurrence and metastasis.^2^ The median overall survival (OS) time for recurrent and/or metastatic (R/M) patients with OSCC is 8–10 months.^3^

Anti‐programmed cell death protein 1 (PD‐1) immunotherapy enhances the immune response to tumors and impairs the growth of cancer cells.^4^ It is rational for the combination between anti‐PD‐1 immunotherapy and chemotherapy for that chemotherapy promotes immunogenic cell death, enhances the release of tumor antigens, and accelerates immune response.^5^ The KEYNOTE‐048 study revealed that pembrolizumab combined with PF regimen chemotherapy (cisplatin/5‐fluorouracil) in the total population improved OS compared with EXTREME regimen (cetuximab plus cisplatin/5‐fluorouracil) for patients with R/M head and neck squamous cell carcinoma (HNSCC).^6^ However, 72% of enrolled patients in the pembrolizumab with PF regimen chemotherapy experienced grade 3 or higher treatment‐related adverse events (TRAEs, defined as adverse medical events potentially related to the treatment that occurs during clinical trial), and 12% patients experienced AEs leading to death.^6^ TPEXTREME trial showed that patients with R/M HNSCC who adopted TPEx (cetuximab, docetaxel, and cisplatin) regimen had no inferior efficacy but fewer AEs (defined as adverse medical events that occurs during clinical trial) of grade 4 or worse (36 vs. 52%) compared with patients with EXTREME (cetuximab, 5­fluorouracil, and cisplatin) regimen,^7^ suggesting a lower toxic chemotherapy regimen in R/M HNSCC. Besides, OSCC is a highly immunodeficient tumor with an immunosuppressive tumor microenvironment.^8^ Docetaxel was reported to convert immunosuppressive tumor microenvironment by facilitating tumor‐associated macrophage to repolarize from M2‐like to M1‐like phenotype and reducing circulating myeloid‐derived suppressor cells (MDSCs).^9^, ^10^ Therefore, TP regimen chemotherapy might be suitable for OSCC patients.

PD­1 ligand 1 (PD­L1) expression was reported to be positively correlated with response to immunotherapy.^11^ Combined positive score (CPS), a variant reflects the PD‐L1 expression, defined as the number of PD­L1‐positive tumor cells and associated immunocytes/the number of total tumor cells×100, predicted prognosis for pembrolizumab therapy in patients with HNSCC.^12^ It suggested that PD­L1‐positive OSCC patients may have a better prognosis by immunotherapy.

Camrelizumab is a high‐affinity PD‐1 antibody that exerts antitumor activity with considerate safety in various types of malignancies.^13^, ^14^ Previous study has preliminarily investigated the efficacy of camrelizumab combined with VEGFR2 inhibitor apatinib in the neoadjuvant therapy for locally advanced resectable OSCC.^15^ However, the efficacy and safety of camrelizumab with chemotherapy in R/M OSCC has never been investigated.

Here, we conducted an open‐labeled, single‐arm, phase Ib trial to explore safety and efficacy of camrelizumab plus TP (docetaxel/cisplatin) regimen chemotherapy as a first‐line therapy for patients with R/M OSCC. We showed that camrelizumab with docetaxel and cisplatin exerted favorite efficacy and tolerable toxicity in patients with R/M OSCC. Besides, we further investigated tumor immune microenvironment in the enrolled patients and found potential predictive biomarkers of immunotherapy in patients with R/M OSCC.

## 2 RESULTS

### 2.1 Clinical characteristic of enrolled patients

**FIGURE 1:**
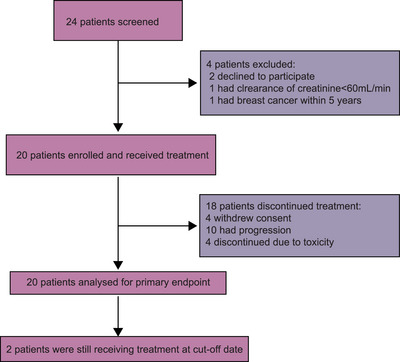
Trial profile of the study.

Between July 14, 2020 and October 8, 2021, 24 patients with recurrent/metastatic oral squamous cell carcinoma (R/M OSCC) were screened, and 20 eligible patients were enrolled. The median follow‐up duration, defined as the interval between the study enrollment and cut‐off date or death, was 18.5 months as of the data cut‐off date (November 1, 2022). Most patients (18 out of 20, 90%) discontinued the treatment due to disease progression (*n* = 10), patient withdrawal (*n* = 4), and AEs (*n* = 4). Among four withdrawn patients, one patient achieved partial response (PR) but requested discontinuation and returned to local hospital for maintenance treatment; three patients were unsatisfied with the treatment effects (efficacy assessed stable disease) and received palliative surgical resection (Figure 1). Participants baseline characteristics are presented in Table 1. Most patients presented with locally recurrent disease (17 out of 20, 85.0%) and HPV‐negative status (19 out of 20, 95%).

**TABLE 1:** Baseline characteristics of enrolled participants.

Characteristics	Participants
Median age, years (range)	59.5 (36–73)
Sex	
Female	9 (45%)
Male	11 (55%)
ECOG performance status	
0	2 (10%)
1	18 (90%)
Smoking history	
Never	10 (50%)
Former	9 (45%)
Current	1 (5%)
Alcohol use	
Never	14 (70%)
Former	5 (25%)
Current	1 (5%)
Primary tumor site	
Tongue	11 (55%)
Gingiva	3 (15%)
Buccal	5 (25%)
Mouth floor	1 (5%)
Recurrence pattern	
Local or reginal recurrence only	17 (85%)
Local or reginal recurrence and distant metastases	1 (5%)
Distant metastases only	2 (10%)
HPV infection	
Positive	1 (5%)
Negative	19 (95%)

### 2.2 Safety and feasibility

All the enrolled patients (100%) experienced at least one TRAE, and nine patients (45%) experienced grade 3 or worse TRAEs (Table 2). The most common TRAEs were nausea (95%), anemia (75%), vomiting (65%), fatigue (65%), stomatitis (60%), RCCEP (60%), neutropenia (40%), and increased alkaline phosphatase levels (40%). The most common grade 3 or worse TRAEs were anemia (15%), stomatitis (15%), neutropenia (10%), and nausea (10%). No treatment‐related deaths occurred during this period. One patient (5%) experienced chemotherapy dose reduction due to toxicity. Two patients (10%) discontinued chemotherapy due to chemotherapy‐related toxicities; of these, one patient continued the treatment with camrelizumab monotherapy, and the other discontinued the treatment permanently.

**TABLE 2:** Adverse events potentially related to the treatment.

Treatment‐related adverse events	Grade 1	Grade 2	Grade 3	Grade 4	Grade 5
Anemia	5 (25%)	7 (35%)	3 (15%)	0 (0%)	0 (0%)
Neutropenia	1 (5%)	5 (25%)	0 (0%)	2 (10%)	0 (0%)
Thrombocytopenia	4 (20%)	2 (10%)	0 (0%)	0 (0%)	0 (0%)
Hypothyroidism	3 (15%)	1 (5%)	1 (5%)	0 (0%)	0 (0%)
Constipation	1 (5%)	0 (0%)	0 (0%)	0 (0%)	0 (0%)
Diarrhea	1 (5%)	6 (30%)	0 (0%)	0 (0%)	0 (0%)
Nausea	7 (35%)	10 (50%)	2 (10%)	0 (0%)	0 (0%)
Stomatitis	4 (20%)	5 (25%)	3 (15%)	0 (0%)	0 (0%)
Vomiting	6 (30%)	6 (30%)	1 (5%)	0 (0%)	0 (0%)
Fatigue	5 (25%)	7 (35%)	1 (5%)	0 (0%)	0 (0%)
Increased alanine aminotransferase	3 (15%)	1 (5%)	0 (0%)	0 (0%)	0 (0%)
Increased aspartate aminotransferase	2 (10%)	0 (0%)	0 (0%)	0 (0%)	0 (0%)
Increased alkaline phosphatase	8 (40%)	0 (0%)	0 (0%)	0 (0%)	0 (0%)
g‐Glutamyltransferase increased	4 (20%)	3 (15%)	0 (0%)	0 (0%)	0 (0%)
Total bilirubin increased	1 (5%)	0 (0%)	0 (0%)	0 (0%)	0 (0%)
Conjugated bilirubin increased	1 (5%)	0 (0%)	0 (0%)	0 (0%)	0 (0%)
Unconjugated bilirubin increased	1 (5%)	0 (0%)	0 (0%)	0 (0%)	0 (0%)
Elevated serum creatinine	2 (10%)	0 (0%)	0 (0%)	0 (0%)	0 (0%)
Pyrexia	2 (10%)	1 (5%)	0 (0%)	0 (0%)	0 (0%)
Reactive cutaneous capillary endothelial proliferation	3 (15%)	9 (45%)	0 (0%)	0 (0%)	0 (0%)
Pruritus	2 (10%)	1 (5%)	1 (5%)	0 (0%)	0 (0%)
Pneumonitis	1 (5%)	4 (20%)	0 (0%)	1 (5%)	0 (0%)
Dermatitis acneiform	0 (0%)	0 (0%)	0 (0%)	1 (5%)	0 (0%)
Rash	1 (5%)	1 (5%)	0 (0%)	0 (0%)	0 (0%)

Potential immune‐related adverse events					
Reactive cutaneous capillary endothelial proliferation	3 (15%)	9 (45%)	0 (0%)	0 (0%)	0 (0%)
Hypothyroidism	3 (15%)	1 (5%)	1 (5%)	0 (0%)	0 (0%)
Pneumonitis	1 (5%)	1 (5%)	0 (0%)	1 (5%)	0 (0%)
Dermatitis acneiform	0 (0%)	0 (0%)	0 (0%)	1 (5%)	0 (0%)

Treatment‐related adverse events (AEs) were defined as adverse medical events potentially related to the treatment that occurs during clinical trial. Grade refers to the severity of the AEs. The criteria of grade was according to the Common Terminology Criteria for Adverse Events (version 5.0). The CTCAE displays Grades 1 through 5 with unique clinical descriptions of severity for each AE based on this general guideline: Grade 1: mild; asymptomatic or mild symptoms; clinical or diagnostic observations only; intervention not indicated. Grade 2: Moderate; minimal, local, or noninvasive intervention indicated; limiting age‐appropriate instrumental activities of daily living (ADL). Grade 3: severe or medically significant but not immediately life‐threatening; hospitalization or prolongation of hospitalization indicated; disabling; limiting self‐care ADL. Grade 4: life‐threatening consequences; urgent intervention indicated. Grade 5: death related to AE.

Thirteen (65%) patients experienced immune‐related AEs (irAEs; defined as adverse medical events potentially related to immunotherapy that occurs during clinical trial) associated with camrelizumab. The most common potential irAEs were RCCEP (60%), hypothyroidism (35%), and pneumonitis (15%). Three patients (15%) had grade 3 or worse potentially irAEs; one patient had grade 3 hypothyroidism; one patient had grade 4 pneumonia during the camrelizumab monotherapy; the patient terminated the treatment and recovered by hormone therapy (methylprednisolone 2 mg/kg/day) within 1 week. Besides, one patient had grade 4 dermatitis acneiform during the camrelizumab with chemotherapy, the patient also terminated the treatment and recovered by hormone therapy (methylprednisolone 2 mg/kg/day) for 2 weeks.

### 2.3 The efficacy and clinical outcome of patients

**FIGURE 2:**
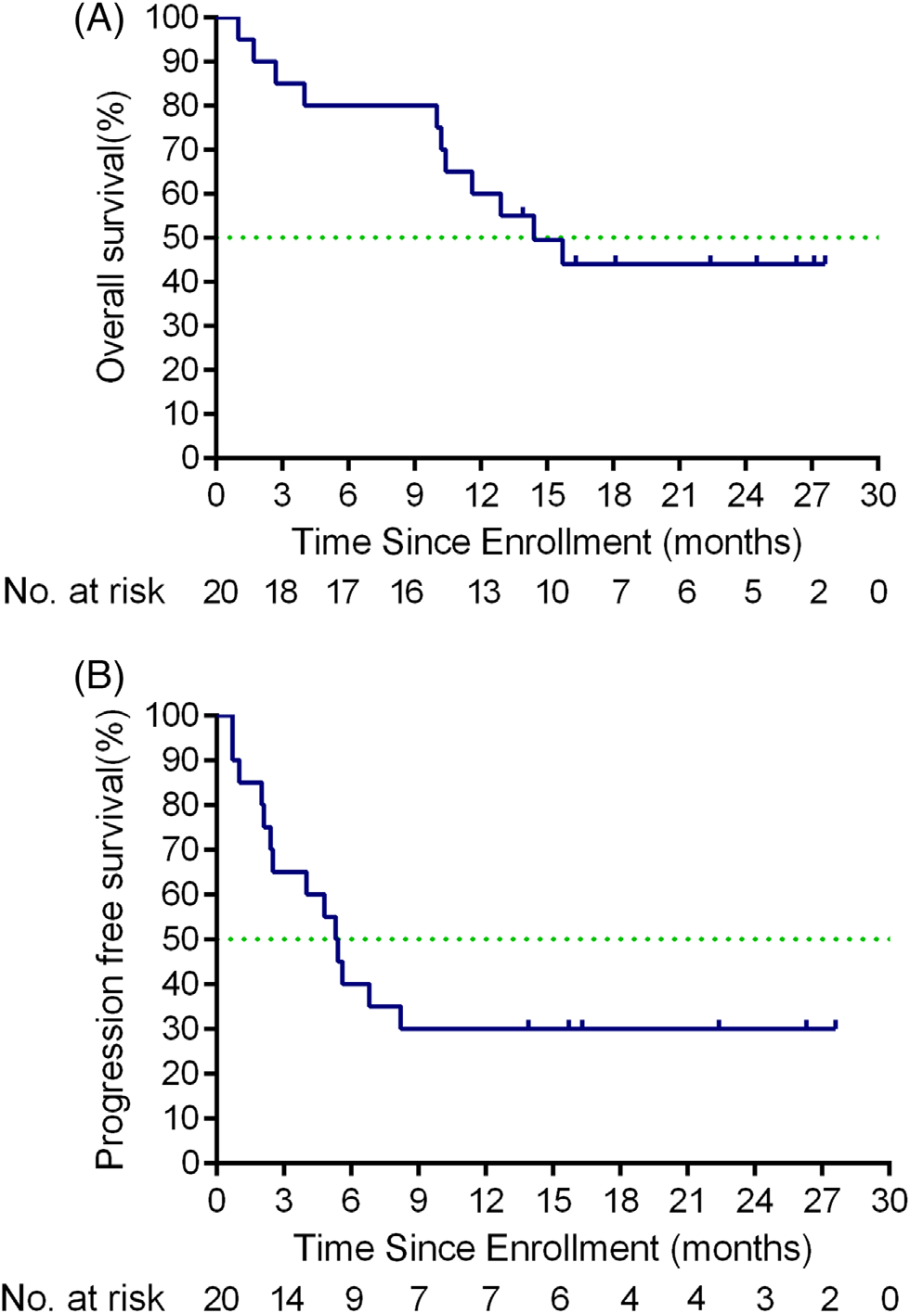
Kaplan–Meier curves of progression‐free survival, and overall survival. (A) Overall survival, and (B) progression‐free survival were assessed in the intention‐to‐treat population (*n* = 20).

**FIGURE 3:**
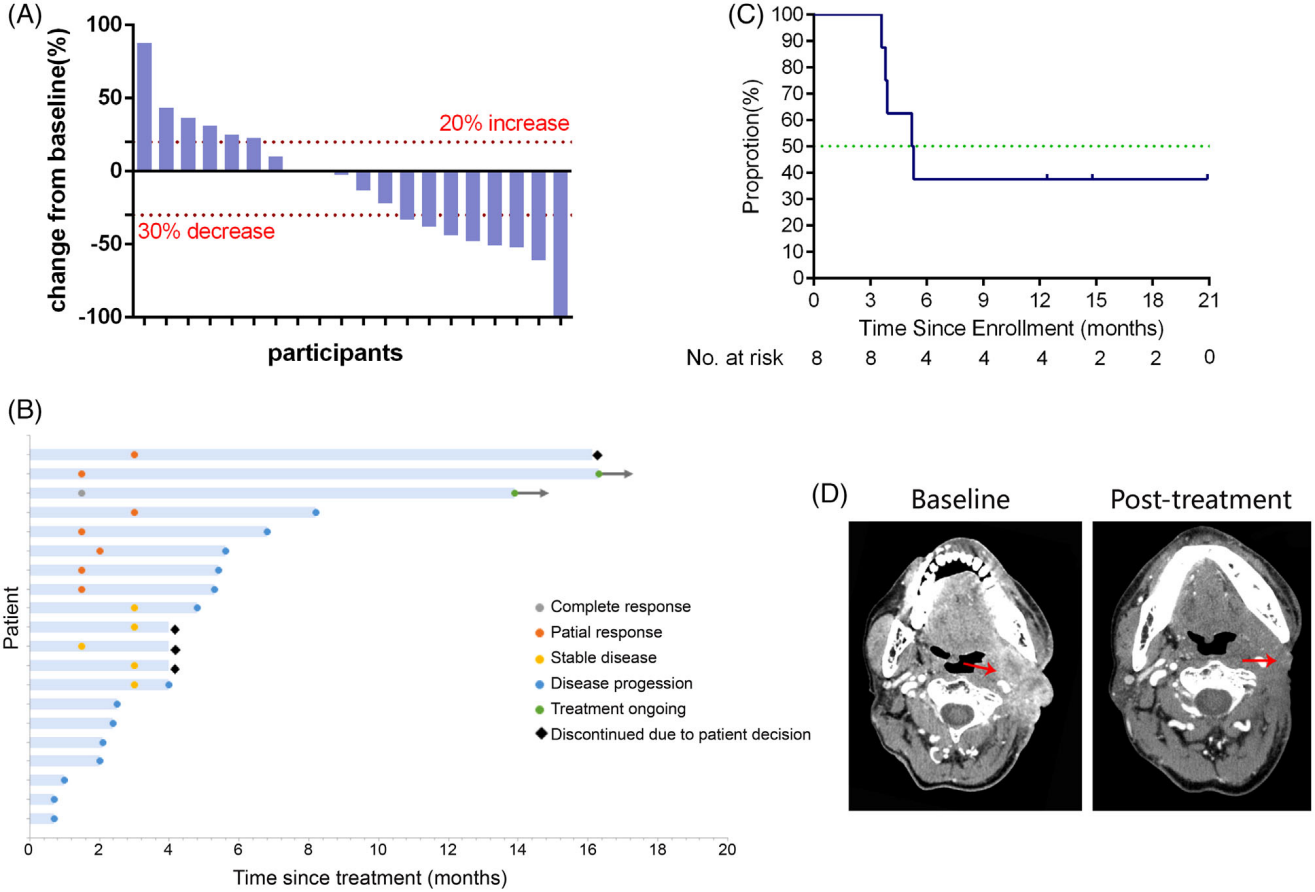
The antitumor efficacy in the efficacy evaluable population (*n* = 20). (A) Best percentage change from baseline in target lesion. The dashed line at −30% change represents the RECIST version 1.1 cutoff to define partial response or complete response, and at +20% change represents the RECIST version 1.1 cutoff to define progressive disease; (B) duration of responses. The length of each bar represents the duration treatment of each patient; (C) duration of response was assessed in the intention‐to‐treat population (*n* = 8); (D) the patient #5 achieved partial response, computed tomography showed the radiographic images at the baseline and posttreatment at 3 months, the red arrows referred as target lesion of the patient #5.

Ten (50%) deaths occurred on the cut‐off date (November 1, 2022). The median OS was 14.4 months (95% CI 8.8−20.0 months) (Figure 2A). The median progression‐free survival (PFS) was 5.35 months (95% CI 4.0−6.6 months) (Figure 2B). Overall, one patient had a complete response (CR), seven patients had a confirmed PR, six patients had confirmed stable disease (SD), and six patients had confirmed progressive disease (PD). The objective response rate (ORR) was 40.0% (eight out of 20; 95% CI 19.1−63.9%), and disease control rate (defined as patients with CR, PR, or SD) was 70% (14 out of 20; 95% CI 45.7−88.1%) (Figures 3A and D). Among the eight responders, the median duration of response (DOR) was 5.25 months (95% CI 3.3−7.1 months) (Figures 3B and C).

### 2.4 Subgroup analyses

**FIGURE 4:**
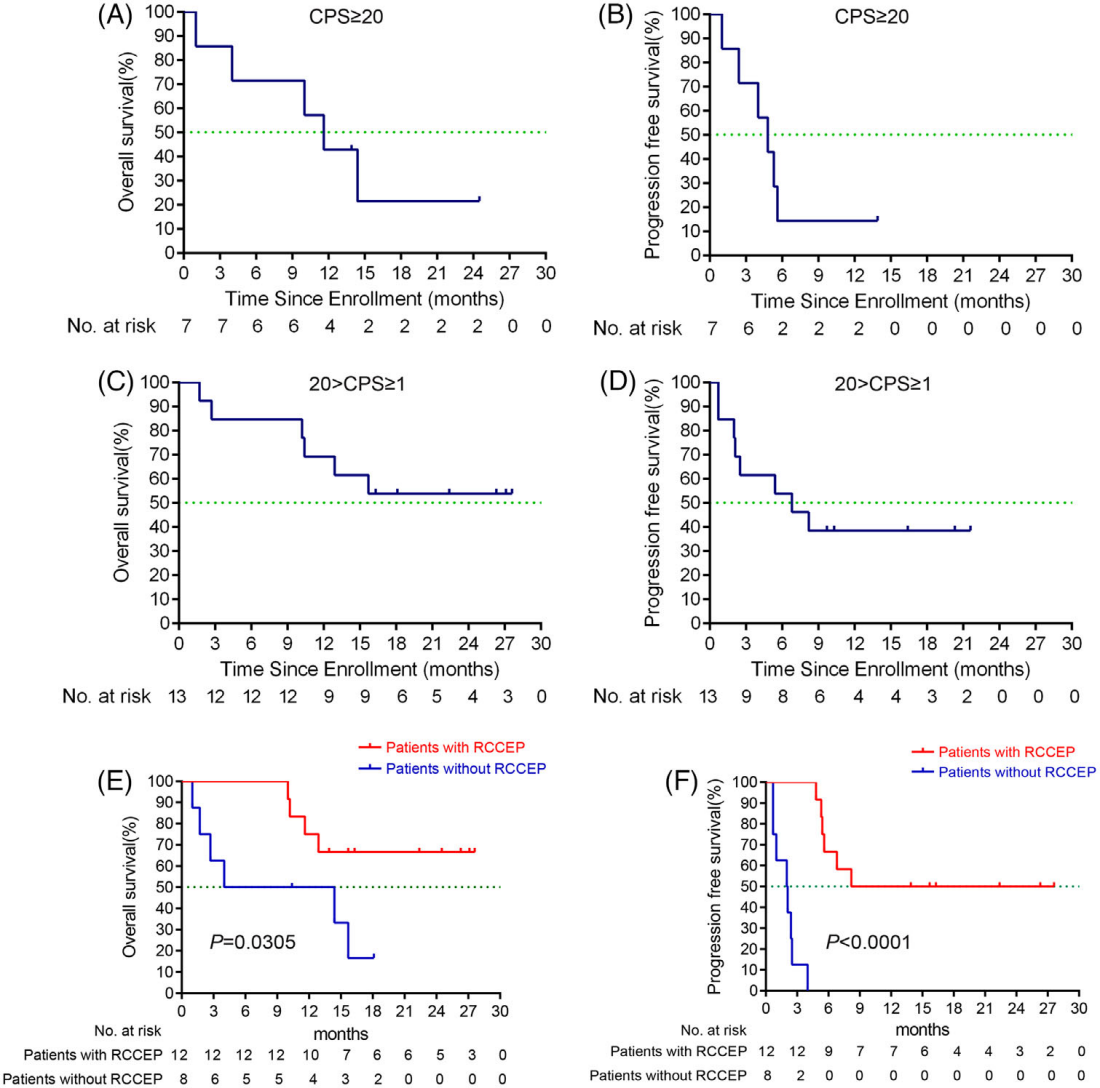
Subgroup analysis (A) overall survival and (B) progression‐free survival were assessed in the PD­L1 CPS ≥ 20 population (*n* = 7); (C) overall survival, and (D) progression‐free survival were assessed in the PD­L1 20 > CPS ≥ 1 population (*n* = 13); (E) overall survival, and (F) progression‐free survival were assessed in patients with RCCEP (*n* = 12) and without RCCEP (*n* = 8). PD‐L1, programmed death ligand 1; RCCEP, reactive cutaneous capillary endothelial proliferation.

In our study, seven patients (seven out of 20; 35%) had a CPS of 20 or more. In the CPS ≥ 20 patients, the median OS was 11.6 months (95% CI 7.5−15.7 months) (Figure 4A); the median PFS was 4.8 months (95% CI 2.7−6.9 months) (Figure 4B), and the overall response rate (ORR) was 42.9% (three out of seven; 95% CI 9.9−81.6%). In the 20 > CPS ≥ 1 patients, the median OS was not reached (Figure 4C); the median PFS was 6.8 months (95% CI 0.1−13.5 months) (Figure 4D), and the ORR was 38.5% (five out of thirteen; 95% CI 13.9−68.4%).

A previous study reported that the incidence of RCCEP correlated with camrelizumab efficacy in patients with advanced hepatocellular carcinoma.^16^ In our study, among 12 patients with RCCEP, eight patients achieved PR or CR, four patients achieved SD, and no patients without RCCEP achieved PR or CR. The occurrence of RCCEP was significantly associated with improved median PFS (17.9 vs. 2.05 months, HR, 0.0159, 95% CI, 0.0031−0.0822, *p* < 0.0001) (Figure 4E) and OS (not reached vs. 9.2 months, HR, 0.2194, 95% CI, 0.0555−0.8673, *p* = 0.0305) (Figure 4F) were observed. Previous study suggested that p16 expression was correlated with the positive expression rate of PD‐L1.^17^ However, as only one patient was p16 positive in our study, the association between p16 status and PD‐L1 expression could not be reached. The patient diagnosed with tongue cancer with lung metastasis but without local recurrence. The patient achieved PR and continued the treatment by the cut‐off date.

### 2.5 The potential biomarkers of immune microenvironment

**FIGURE 5:**
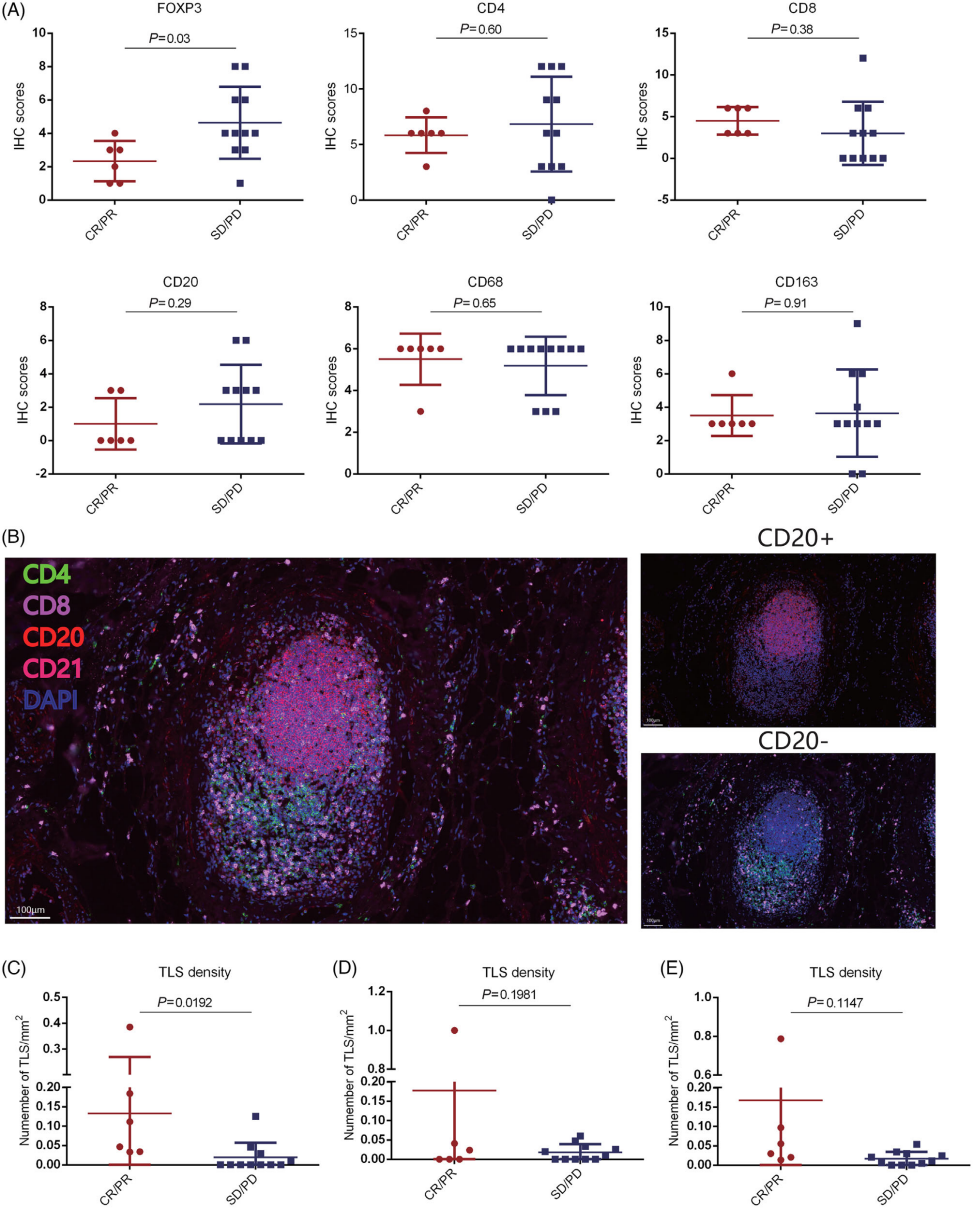
The association between efficacy and immune microenvironment in the enrolled patients (A) the expression of FOXP3, CD4, CD8, CD68, CD163 and TGFβ1 were evaluated immunohistochemistry (IHC), the association between the expression of FOXP3, CD4, CD8, CD68, CD163, TGFβ1, and response (CR/PR(*n* = 6) vs. SD/PD(*n* = 11)) were shown; (B) tertiary lymphoid structures (TLS) were stained by multiplex immunofluorescence using markers DAPI, CD4, CD8, CD20 (B cells), and CD21, presentative images at original magnification were shown; TLS density was counted by the number of TLS per mm^2^ in core tumor area (intratumor TLS), invasive margin of tumor area (peritumor TLS) and total area of tissue section (total TLS), the correlation between intratumor TLS (C), peritumor TLS (D), total TLS (E), density and response (CR/PR(*n* = 6) vs. SD/PD(*n* = 11)) was evaluated.

Baseline tumor sections were eligible from 17 enrolled patients to analyze the tumor immune microenvironment. The expression of FOXP3 was significantly higher in nonresponders (patients with SD/PD) than responders (patients with CR/PR) (Figure 5A). However, no significant differences were observed in the expression of CD4, CD8, CD68, CD163 and TGFβ1 between responders and nonresponders (Figure 5A). For tertiary lymphoid structure (TLS) analysis, patients with higher TLS density had significantly higher response rate in intratumor TLS (defined as TLS in core tumor area) than those with lower TLS density (Figures 5B and C). However, there was no significant correlation between peritumoral (defined as TLS in invasive margin of tumor, which was more than 0.5 mm adjacent to the tumor; Figure 5D) or total (Figure 5E) TLS and response rate.

## 3 DISCUSSION

Our study provided the first evidence that camrelizumab combined with TP regimen chemotherapy was well tolerated and indicated potentially improved median OS, PFS, and ORR in the first‐line treatment of patients with R/M OSCC compared with the efficacy of cetuximab and/or chemotherapy reported by previous studies.

Patients with R/M HNSCC have been shown to benefit from PD‐1 inhibitor therapy. The KEYNOTE‐040 and CheckMate 141 studies have indicated that patients with R/M HNSCC obtained survival benefit by receiving pembrolizumab or nivolumab as second‐line therapy compared with standard second‐line therapy.^18^, ^19^ Currently, cisplatin/5‐fluorouracil (PF) and docetaxel/cisplatin are the preferred chemotherapy regimens for patients with R/M HNSCC. Nevertheless, chemotherapy in PF regimen seemingly exerted a higher toxicity than TP regimen. In the KEYNOTE‐048, 85% of patients receiving pembrolizumab plus PF regimen chemotherapy experienced grade 3 or higher AEs, and 12% of patients died due to AEs.^6^ E1395 trial showed no survival difference between cisplatin plus paclitaxel and cisplatin plus fluorouracil in patients with advanced head and neck cancer but a significantly lower incidence of grade 3 or more AEs (80 vs. 97%) in the cisplatin plus paclitaxel group.^3^ The TPEXTREME trial showed no survival difference (median OS, 14.5 vs. 13.4 months; HR 0.89 [95% CI 0.74−1.08]; *p* = 0.23) but a lower incidence of grade 3 or higher AEs (81 vs. 93%, *p* < 0.0001) in patients with R/M HNSCC administered cetuximab, docetaxel, and cisplatin compared with cetuximab, 5­fluorouracil, and cisplatin.^9^ As a result, we adopted a docetaxel/cisplatin‐based regimen as the chemotherapy in this study. In our study, 50% of patients experienced grade 3 or higher TRAEs, suggesting a possibly less toxic chemotherapy regimen compared with pembrolizumab plus PF regimen chemotherapy in R/M OSCC.

Several studies have reported that irAEs are potentially associated with tumor response in patients receiving immune checkpoint blockade.^20^ RCCEP, a special and most common irAE only observed in patients treated with camrelizumab, was a skin lesion manifested as a red spot or nodules that tended to rupture and bleed.^21^ Wang et al.^22^ reported that RCCEP occurrence was positively associated with better mPFS, mOS, and ORR of camrelizumab in patients with advanced hepatocellular carcinoma. Our results also found significant association between the occurrence of RCCEP and better ORR, PFS, and OS benefit, suggesting that RCCEP might act as an independent prognostic factor of camrelizumab treatment in patients with R/M OSCC. Among those camrelizumab nonspecific irAEs, the most common irAE was hypothyroidism, which occurred in 35% participants. This result was also similar with the previously reported irAEs not only for camrelizumab,^23^, ^24^ but also for pembrolizumab and nivolumab.^21^, ^25^

The KEYNOTE‐048 trial revealed that pembrolizumab plus PF regimen chemotherapy (platinum/5‐fluorouracil) prolonged median OS in the total population and in CPS ≥ 1 population compared with standard first‐line treatment in patients with R/M HNSCC. However, primary site of HNSCC patients would affect the efficacy of anti‐PD1 immunotherapy. Kate Clancy et al.^26^ reported that R/M OSCC patients have a poorer OS than oropharyngeal and laryngeal patients who receiving pembrolizumab or nivolumab. Numerous studies have found that anti‐PD1 immunotherapy could be enhanced in patients with high PD‐L1 expression in the tumor microenvironment in certain malignancies, including HNSCC.^27^, ^28^ In our study, the median was 14.4 months (95% CI 9.9−18.9 months) in CPS ≥ 1 patients with R/M OSCC who were administered camrelizumab plus docetaxel and cisplatin as first‐line therapy, suggesting significant survival benefit in CPS ≥ 1 population with R/M OSCC compared with cetuximab and/or chemotherapy. Furthermore, the median PFS was 5.35 months (95% CI 4.0−6.6 months), and the ORR was 40.0% (95% CI 19.1−63.9%), indicating a similar antitumor activity with pembrolizumab plus PF regimen chemotherapy.

In the KEYNOTE‐048 study, pembrolizumab plus PF regimen chemotherapy improved the median OS to 14.7 months, median PFS to 5.8 months, and ORR to 43% in patients with R/M HNSCC having a CPS ≥ 20.^6^ In our study, the median OS was 14.4 months, median PFS was 5.3 months, and ORR was 42.9%, indicating camrelizumab plus docetaxel/cisplatin chemotherapy had similar antitumor activity in patients with R/M OSCC having a CPS ≥ 20 compared with pembrolizumab plus PF regimen chemotherapy. Furthermore, we investigated the 20 > CPS ≥ 1 population, showing a favorite median OS, PFS, and ORR. Our result indicates camrelizumab plus docetaxel/cisplatin chemotherapy could also exert therapeutic effect in 20 > CPS ≥ 1 patients with R/M OSCC.

HPV status was reported to be associated with the positive expression rate of PD‐L1.^17^ In our study, the patient with p16 positive showed a favorite efficacy to the treatment. However, with the overall small sample size, the association between HPV status and PD‐L1 expression level could not be reached, and it deserves further investigation in the future.

In our study, we observed higher expression of FOXP3^+^ cells infiltration in responders at baseline, indicates higher infiltration of Tregs may affect the response therapeutic effect in patients with R/M OSCC. Consistent with previous studies, CD4^+^ T cells, CD8^+^ T cells, CD68^+^, CD163^+^, and TGFβ1^+^ cells showed no significant correlation with the response rate at baseline. Recently, TLS was identified as a predictive biomarker of immunotherapy, but the location of TLS affected the response to immunotherapy distinctively. Our results found a positive association between density of intratumor TLS and response to camrelizumab with docetaxel/cisplatin treatment, suggested that TLS localized to the core of tumor might predict the efficacy of immunotherapy.

Our study had several limitations, including a single‐arm study design and a relatively small sample size. Additionally, the adoption of an open‐label, single‐center study needs confirmation by a multicenter, randomized, double‐blind trial. Additionally, the study was underpowered for the subgroup analysis of efficacy based on PD‐L1 status and RCCEP occurrence.

In conclusion, camrelizumab plus docetaxel and cisplatin as first‐line therapy demonstrated tolerable TRAEs occurrence and potentially favorable antitumor activity in patients with R/M OSCC. Our data suggest that camrelizumab plus TP‐regimen chemotherapy is a promising first‐line therapy for R/M OSCC, and further a large scale randomized, double‐blind trials are required.

## 4 MATERIALS AND METHODS

### 4.1 Study design

The CHANCE study was an open‐label, single‐arm, phase Ib trial conducted at Shanghai Ninth People's Hospital. The inclusion criteria for eligible patients were: aged 18–75 years; histologically diagnosed as R/M OSCC with no surgical indication; had sufficient tumor sample for PD­L1 and HPV status (HPV‐positivity was defined as tumors that were p16 positive by immunohistochemistry (IHC) staining (clone E6H4; CINtec histology kit; Roche diagnostics)) testing; CPS ≥ 1; had measurable lesion according to Response Evaluation Criteria in Solid Tumors (RECIST) version 1.1^29^; had an score of Eastern Cooperative Oncology Group (ECOG) ≤ 1; had a clearance of serum creatinine > 60 mL/min to receive cisplatin exposure; and had acceptable bone marrow, cardiac, liver, and renal functions. Exclusion criteria included previous systemic therapy for HNSCC after metastasis or recurrence; had surgical operation or radiotherapy within 6 weeks before screening; had a cumulative dose of cisplatin > 300 mg/m^2^; had a history of other malignancies within 5 years before study entry, except for cured skin basal cell carcinoma, skin squamous cell carcinoma, carcinoma in situ of the cervix and early prostate cancer; had active or a history of autoimmune disease; administrated immunosuppressive drugs or systemic hormone therapy with a dose of 10 mg or more per day, and still continuing within 2 weeks before screening; previously treated with CTLA‐4, PD‐1, or PD‐L1 antibodies; active or uncontrollable brain metastases; human immunodeficiency virus or treponema pallidum infection; persistent or active infection requiring intervening; and active pulmonary disease (asthma, obstructive pulmonary disease, and interstitial pneumonia) or had a history of active pulmonary tuberculosis.

The protocol of trial was reviewed and approved by the ethics committee of the Shanghai Ninth People's Hospital (SH9H‐2019‐T354‐3), and the trial was done following the guidelines of Declaration of Helsinki and Good Clinical Practice. Written informed consents were signed before study entry. The study was registered in the Chinese Clinical Trial Registry (ChiCTR1900026736) and also registered in ClinicalTrials.gov (NCT05611463).

### 4.2 Procedures

Patients were administered camrelizumab (200 mg) on day 1 with docetaxel (75 mg/m^2^) and cisplatin (75 mg/m^2^) on day 2 every 3 weeks for a maximum of six cycles, then followed by camrelizumab monotherapy every 3 weeks. The dose of camrelizumab based on the phase I trial of camrelizumab in solid tumors.^21^ The treatment continued until disease progression, intolerable toxicity, or patient‐initiated withdrawal. Participants with unconfirmed disease progression that clinically stable could continue the treatment until progression was confirmed by imaging 4 weeks later. Camrelizumab was paused if grade 2 of TRAEs occurred and continued until the grade of TRAEs dropped to 0 or 1 within 12 weeks. Camrelizumab was permanently discontinued if grade 3 or worse TRAEs occurred or grade 2 of TRAEs failed to recover to 0 or 1 within 12 weeks. The dose of docetaxel and cisplatin was allowed to reduce to 60 mg/m^2^ if grade 3 or worse TRAEs occurred in the last cycle and recovered to grade 0 or 1 within 6 weeks, and permanently discontinued if failed to recovered to grade 0 or 1 within 6 weeks.

Tumor imaging was evaluated by using computed tomography and magnetic resonance imaging. The assessment was conducted at baseline, every two cycles through the first year since study entry, and every 3 months since the second year. The response to treatment was determined according to the revised Response Evaluation Criteria in Solid Tumors (RECIST version 1.1),^30^ and toxicity was recorded each week during and after the therapy in accordance with the Common Terminology Criteria for Adverse Events (version 5.0). Survival information was obtained from participants every 3 months during follow‐up.

### 4.3 IHC and multiplex immunofluorescence analysis

Baseline tumor biopsy was obtained from metastatic or recurrent lesions within 6 months before enrollment. CPS, measured by using the PD­L1 IHC 22C3 assay (Agilent Technologies, Santa Clara, CA, USA), was adopted to evaluate PD­L1 expression. We defined CPS ≥ 1 as PD‐L1 positive.

The tumor‐infiltrating lymphocytes (TIL) were analyzed by IHC assay for CD4 (Servicebio; catalog number GB15064, 1:200 dilution), CD8 (Servicebio; catalog number GB12068, 1:1500 dilution), TGFβ1 (Servicebio; catalog number GB11179, 1:500 dilution), CD68 (Servicebio; catalog number GB113109, 1:200 dilution), CD163 (Servicebio; catalog number GB113152, 1:500 dilution), and FOXP3 (Servicebio; catalog number GB11093, 1:500 dilution) markers. Then, the sections were scanned and scored by proportion of positive cells multiplied by staining intensity. The definition of proportion of positive cells and staining intensity were described in our previous study.^31^ Briefly, the grade of positive cells proportion was defined as follows: <10% (grade 0), 10–25% (grade 1), 25–50% (grade 2), 50–75% (grade 3), and 75% (grade 4). The grade of staining intensity was defined as follows: 0 (no staining), 1 (bright yellow), 2 (orange), and 3 (brown). TLS was accessed multiplex immunofluorescence. Primary rabbit anti‐human CD4 antibody (Servicebio; catalog number GB15064, 1:200 dilution), primary mouse anti‐human CD8 antibody (Servicebio; catalog number GB12068, 1:1500 dilution), primary mouse anti‐human CD20 antibody (Servicebio; catalog number GB14030, 1:300 dilution), and rabbit anti‐human CD21 antibody (Servicebio; catalog number GB14031, 1:300 dilution) were utilized for TLS analysis. TLS was defined as CD20, CD4, CD8, and CD21 enriched area. The density of intratumor TLS (number of TLS per mm^2^ in core tumor area), peritumor TLS (number of TLS per mm^2^ in invasive margin of tumor area), and total TLS (number of TLS per mm^2^ in total area of tissue section) was evaluated, respectively.

### 4.4 Outcomes

The primary endpoint was occurrence of grade ≥ 3 TRAEs, defined as the proportion of participants with grade 3 or worse TRAEs. TRAEs were collected to access safety and tolerability during the treatment period and 1 month after treatment withdrawal or 3 months if serious adverse events occurred. TRAEs were graded according to the National Cancer Institute Common Terminology Criteria for Adverse Events (NCI‐CTCAE) version 5.0.^22^ Secondary endpoints for the study were OS, defined as time between study enrollment and death from any cause; ORR, defined as the proportion of participants with PR or CR according to RECIST (version 1.1) assessed by a specialized radiologist and confirmed by two other masking radiologists; PFS, defined as time between enrollment and the first disease progression or death from any cause, whichever occurred first; and DOR, defined as interval from the first time participants achieve CR or PR to the first evaluation of PD.

### 4.5 Statistical analysis

Sample size was calculated by performing PASS version 14.0.3. The trial was assumed that 72% patients with R/M OSCC receiving PF (cisplatin+5‐fluorouracil) regimen chemotherapy experienced grade 3 or worse TRAEs.^3^ The occurrence of grade 3 or worse TRAEs was assumed to decline 1/3 to 48% with the therapy of camrelizumab to TP regimen chemotherapy. Patients were accrued for 24 months and followed up for an additional 18 months. The study designed a power of 80% to detect a difference, and a 10% (one‐sided) type I error rate. We calculated a minimum sample size of 18 patients, and we planned to accrue 20 patients by assuming a 10% drop‐out rate. The secondary endpoints (OS, PFS, and DOR) were analyzed by Kaplan–Meier method with corresponding 95% CIs for the median estimation. Subgroup analyses including the occurrence of reactive cutaneous capillary endothelial proliferation (RCCEP) and CPS PD‐L1 ≥ 20 were accessed by performing Mann–Whitney test. The correlation between TIL, TLS, and response was analyzed by Student's t‐test. *p* Value < 0.05 was defined as statistically significant. The cut‐off date for this study was November 1, 2022.

## AUTHOR CONTRIBUTION

Guoxin Ren and Jingzhou Hu designed the study. Houyu Ju, Dongliang Wei, and Qi Ding obtained and interpreted the data. Houyu Ju, Mengyu Rui, and Yunteng Wu drafted the manuscript. Yang Liu and Zongyu Fan performed statistical analysis. Yanli Yao and Guoxin Ren revised the manuscript. All authors read and approved the final manuscript.

## CONFLICT OF INTEREST STATEMENT

All the authors have no potential conflict of interest.

## ETHICS STATEMENT

This study was registered in the Clinical Trial Registry (NCT05611463) and approved by the Institutional Review Board of Shanghai Ninth People's Hospital affiliated to Shanghai Jiao Tong University School of Medicine. The study was conducted in accordance with the Declaration of Helsinki and Good Clinical Practice. Written informed consents were obtained from all patients before participated.

## Data Availability

Data are available upon reasonable request to the corresponding author.
